# High-resolution records of cesium, plutonium, americium, and uranium isotopes in sediment cores from Swiss lakes

**DOI:** 10.1007/s11356-022-20785-y

**Published:** 2022-05-20

**Authors:** Stefan Röllin, José Antonio Corcho-Alvarado, Hans Sahli, Victoria Putyrskaya, Eckehard Klemt

**Affiliations:** 1 Federal Office for Civil Protection, Spiez Laboratory, Spiez, Switzerland; 2 Medizin Campus Bodensee, Klinikum Friedrichshafen, Friedrichshafen, Germany; 3 Hochschule Ravensburg-Weingarten, University of Applied Sciences, Weingarten, Germany

**Keywords:** Lake sediments, Pu, U, Am, Isotopic ratios, ICP-MS, Nuclear weapon testing, Chernobyl, Non-global fallout, Pu age

## Abstract

The Aare river system in Switzerland, with two nuclear power plants on the banks of the river, and its intermediate lakes and reservoirs, provides a unique opportunity to analyze the contribution of different sources to the radioactive contamination. Sediment cores were collected from two lakes and a reservoir, all connected by the river Aare. In order to study the influence of the Chernobyl accident, one sediment core was collected from a lake in the southern part of Switzerland. The sediment cores were sliced and analyzed with gamma ray spectrometry. Plutonium, americium, and uranium were extracted radiochemically, and their concentrations were measured with a sector field ICP-MS. The uranium isotope ratios were further measured with a multi collector ICP-MS. The maximum ^137^Cs activity from the Chernobyl accident and the Pu and ^137^Cs activities associated to the 1963 global fallout maximum were well identified in sediments from all three lakes. High-resolution records of plutonium isotopes in the zone of the sediments corresponding to the period of maximum fallout from the atmospheric nuclear weapon testing showed distinct fingerprints, depending on the different test activities. Pu isotope ratios could be used to detect non-global fallout plutonium. The ratio ^241^Am/^241^Pu was used to determine the age of the plutonium. Despite of very low ^241^Pu and ^241^Am concentrations, the calculated plutonium production dates seemed to be reasonable for the sediment layers corresponding to the NWT tests. The calculated production date of the plutonium in the upper most 15 cm of the sediment core seemed to be younger. The reason for this could be additional non-global fallout plutonium. For the lake sediments, natural ratios for ^235^U/^238^U and enriched or depleted ratios for ^234^U/^238^U were measured, depending on the lake. A small increase of the ^236^U/^238^U ratio could be recognized for the NWT zone in all three lakes and, for Lake Lugano, a further distinct increase in the Chernobyl layer.

## Introduction

The Aare river system in Switzerland, with two nuclear power plants on the banks of the river, and its intermediate lakes and reservoirs, provides a unique opportunity to analyze the contribution of different sources to the radioactive contamination. Dating of sediment layers can help to identify different radioactive contamination sources. At a global scale, the major source of cesium-137 and plutonium (Pu) in the environment is by far the atmospheric radioactive fallout originated from the nuclear weapon tests (NWT). The amount of global fallout (GF) depends on the latitude and the amount of precipitation. ^137^Cs deposition from the Chernobyl nuclear power plant accident in 1986 is more heterogeneous than the global fallout and was mainly influenced by precipitation events that occurred late in April and early in May 1986, when the radioactive cloud travelled across the European continent (Meusburger et al. (2020)). Meusburger et al. (2020) calculated the amount of ^137^Cs from the global fallout in soil samples from the amount of Pu under the assumption of a constant Pu to ^137^Cs ratio for the global fallout. The difference to the total amount of ^137^Cs was then attributed to the deposition from the Chernobyl accident. This resulted in a comprehensive survey of the ^137^Cs contamination in Europe from the Chernobyl accident. Depth profiles of Pu and ^137^Cs in sediments can also be used to distinguish between the two main inputs. ^137^Cs fallout has a distinct activity maximum in 1986 from Chernobyl and another one in 1963 from the NWT, whereas plutonium often only has a distinct activity maximum in 1963, except for sediments close to Chernobyl. These maxima have been identified in many proxy records worldwide (e.g., sediment and ice cores, corals). These features are nowadays well-established time markers, and they are commonly used for validating ^210^Pb chronologies in sedimentation studies (Ketterer et al. (2004a), Gabrieli et al. (2011), Putyrskaya et al. (2015), Wang et al. (2020), Sanchez-Cabeza et al. (2021)).

It is known that due to the differences in weapon design and the parameters of the nuclear weapon tests realized, the ^240^Pu/^239^Pu ratio in global fallout has as well varied with time. Koide et al. (1985) were the first to propose the use of time features of the ^240^Pu/^239^Pu atomic ratio as time markers. In their study of dated polar ice cores from Antarctica and Greenland, pre-moratorium period (< 1959) was characterized by higher ^240^Pu/^239^Pu ratios than the post-moratorium period (> 1961). Records of the ^240^Pu/^239^Pu atom ratio in an alpine ice core (Mont Blanc, France) and in annual grass samples from Rothamsted, UK (Warneke et al. (2002)) showed similar patterns to the ones observed by Koide. The distinct features in the ^240^Pu/^239^Pu atomic ratio were associated to certain weapon-test periods.

As plutonium from the Chernobyl nuclear power plant accident was contained in the non-volatile fraction of the nuclear fuel debris, it was deposited mainly in the surroundings of the reactor and in Eastern and Northern Europe, whereas the major part of Europe did not receive measurable levels of Pu from the Chernobyl fallout (Meusburger et al. (2020)). Mietelski and Was (1995) calculated the Chernobyl plutonium fraction in forest litter samples in Poland, assuming a constant activity ratio of ^238^Pu to the sum of plutonium (^239^Pu + ^240^Pu) for the global fallout and Chernobyl plutonium. Ketterer et al. (2004b) used atomic ratios of plutonium isotopes to recognize and distinguish non-fallout sources.

Uranium isotope ratios can also be an important signature for the evidence of additional contamination of environmental samples with low amounts of irradiated uranium from nuclear reactors (Boulyga and Heumann (2006)). ^236^U is produced in irradiated fuel and can be used to distinguish between natural and irradiated uranium. In natural uranium, extremely small amounts of ^236^U from cosmic ray activation processes lead to isotope ratios of ^236^U/^238^U < 6.10^−10^ (Richter et al. (1999)). The ratio ^236^U/^238^U in environmental samples depends on the ratio of the amount of artificial to natural uranium. Isotope ratios were widely used to differentiate between natural uranium and uranium resulting from depleted uranium ammunition (Danesi et al. (2003), UNEP (2001)). Natural geochemical processes can also change natural isotope ratios in rocks (Rosholt (1983), Suksi (2001)) and sediments (Thollon et al. (2020)).

Here, we present high-resolution records of Cs, U, Pu, and Am activities and atom ratios obtained in sediment cores from three Swiss lakes (Lake Lugano, Lake Brienz, and Lake Biel) and a fresh water reservoir (Klingnau reservoir). Lake Lugano is situated in the southern part of Switzerland, a region known to have been heavily affected by the Chernobyl fallout (Huber et al. (1996)). The two other lakes and the reservoir, in the northern part of Switzerland, are connected by the river Aare. Besides radioactive contamination due to nuclear weapon testing and the Chernobyl accident, radioactive discharges from the two nuclear power plants (Mühleberg and Beznau) and a nuclear facility (Paul Scherrer Institute, PSI) have influenced the contamination of the sediments. The ^240^Pu/^239^Pu atom ratio and Pu activity concentrations were used as time markers for validating sediment chronologies and to distinguish non-global plutonium. The age of the Pu is a valuable information for the determination of the origin of the contamination. Despite the very low Pu concentrations and the possibility of different migration, Pu age determinations performed with the mother/daughter pair ^241^Pu and ^241^Am led to reasonable ages. Uranium isotope ratios and ^236^U /^239^Pu isotope ratios can also be useful to characterize a contamination with nuclear material. Although the concentrations of the artificial ^236^U isotope were very low, it could still be analyzed in all lake sediments with a multi-collector ICP-MS.

### Materials and methods

#### Study sites

**Fig. 1:**
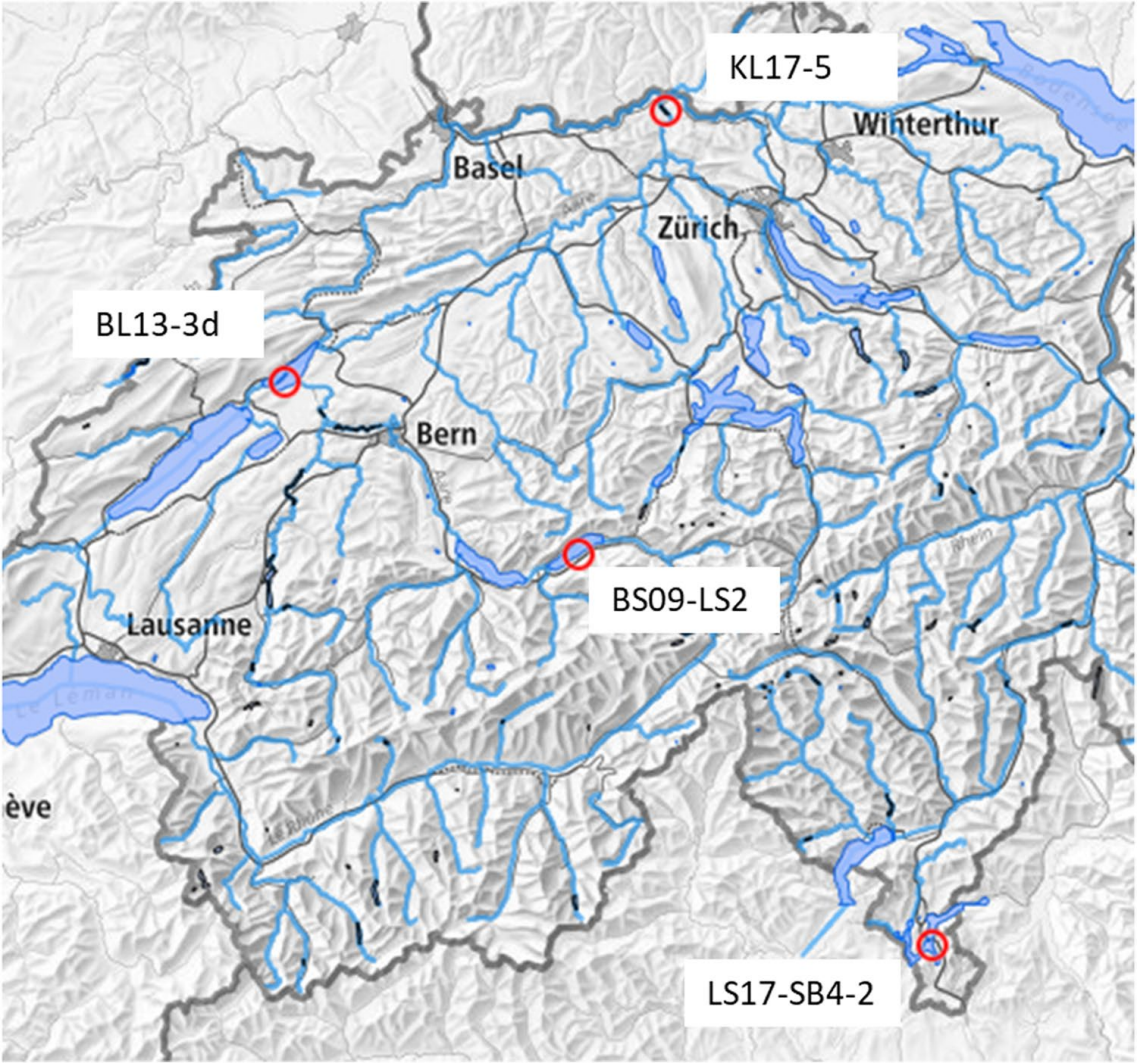
Map of the four sampling positions of Lake Lugano (LS17-SB4-2), Lake Brienz (BS09-LS2), Lake Biel (BL13-3d) and Klingnau Reservoir (KL17-5). The map was reproduced with the tool from the Federal Office of Topography swisstopo

Figure 1 shows a map of Switzerland with the four sampling positions.

Lake Lugano is situated in the southern part of Switzerland, on the border between Switzerland and Italy. The lake is divided into two main basins (northern and southern) which are separated by an artificial dam built in 1844. After the Chernobyl accident in 1986, roughly 24 kBq m^−2^ of ^137^Cs were deposited onto the surface of Lake Lugano (Santschi et al. (1990)). The sediment core (LS17-SB4-2) was taken in 2017 from the southern basin of Lake Lugano (near Melide).

Lake Brienz, Lake Biel, and the Klingnau Reservoir are all connected by the river Aare, one of the major rivers in Switzerland. The river comes from glaciers in the Grimsel area and ends in the north of the country in the confluence with the River Rhine. The sediment core from Lake Brienz (BS09-LS2) was taken in 2009 on the left side of the lake near Iseltwald. After passing the Lake of Thoune, the Aare River flows into the Lake of Biel as a result of artificial channels built at the end of the nineteenth century (Albrecht et al. (1999)). The nuclear power plant Mühleberg is situated on the banks of the River Aare, upstream of Lake Biel. The boiling water type nuclear reactor started operation in 1971 and uses water from the River Aare for cooling. The sediment core from Lake Biel (BL13-3d) was taken in 2013, near Lüscherz. The Klingnau Reservoir is an artificially dammed reservoir located just before the confluence of the Aare with the Rhine. It was built in 1935 because of the construction of the Klingnau dam and a hydroelectric power plant. This reservoir is situated about 120 km downstream of the Mühleberg nuclear power plant, and 7–10 km downstream of the Beznau nuclear power plant and the PSI research nuclear facility. The sediment core from Klingnau Reservoir (KL17-5) was taken in 2017 on the left bank near Kleindöttingen.

Table 1 shows the major characteristics of the four sediment cores.

**Table 1:** Major characteristics for the four sediment cores

Object	Core ID	Coordinates WGS84	Sampling date	Core length, cm	Lake/reservoir depth, m	Sedimentation rate, cm/year
Lake Lugano	LS17-SB4-2	N 45.93968° E 8.95832°	03.05.2017	72	84	0.3–0.5
Lake Brienz	BS09-LS2	N 46.71683° E 7.97476°	25.05.2009	66	50	0.3–0.4
Lake Biel	BL13-3d	N 47.05359° E 7.13611°	18.07.2013	135	55	0.7–0.9
Klingnau Reservoir	KL17-5	N 47.58255° E 8.23538°	02.11.2017	136	0.2	1–5

#### Sampling and sample preparation

Sediment cores were collected using gravity corers with an inner diameter of 59 mm for Lake Biel, Lake Brienz, and Klingnau Reservoir, and an inner diameter of 58 mm for Lake Lugano. The sediment cores were stored at 4 °C in a refrigerator before they were opened. They were split longitudinally, photographed, and sliced into 1-cm layers. After freeze-drying and homogenizing by grinding, each sediment sample was put into plastic beakers and measured with gamma spectrometry. Detailed information of the sampling and gamma measurements are reported elsewhere (Putyrskaya et al. (2015), Putyrskaya et al. (2020)). After the gamma analysis, the sediment samples were ashed at 520 °C. Up to 5 g of aliquots of ashed material was used for radiochemical analysis, as described elsewhere (Röllin et al. (2009), Sahli et al. (2017)). For determining the plutonium isotope activities, a known amount of ^242^Pu (3 pg) was added as a radiochemical recovery tracer. ^115^In was added as an internal standard to determine the ^238^U isotope concentration by external calibration. The samples were digested by borate fusion. The melt was dissolved in 175 ml 4.5 M HNO_3_ and after silicates were precipitated, 1.4 ml aliquots were filtered and diluted for quantitative analysis of ^238^U, as reported elsewhere (Sahli et al. (2017)). After filtration of the silicates, Pu and U radionuclides were separated and purified using TEVA and UTEVA extraction chromatography resins, as described elsewhere (Röllin et al. (2020)). This separation procedure was extended for ^241^Am analysis, by coupling a DGA cartridge after the UTEVA column. To the breakthrough from the TEVA column, 3 pg of ^243^Am tracer was added. Matrix elements were washed out with 100 ml 2% HNO_3_. The Am fraction was eluted with 20 ml 0.5 M HCl, evaporated to dryness and then dissolved in 20 ml 2% HNO_3_.

#### Mass spectrometry measurement

Mass spectrometric measurements were performed on a sector-field ICP-MS (Element 2, Thermo Fisher Scientific) and a multi-collector ICP-MS (Neptune plus, Thermo Fisher Scientific). Samples were introduced with a PFA-ST nebulizer (Elemental Scientific Incorporation).

For quantitative analysis of ^238^U, the 1.4 ml aliquots of the dissolved samples were diluted in 40 ml 2% HNO_3_. These solutions were further diluted 1:100 with 2% HNO_3_/0.002% HF in order to avoid any matrix suppression. ^238^U was determined with an external calibration in the expected concentration range. For the analysis of plutonium isotopes and ^241^Am, the corresponding eluates were introduced with an Apex nebulizing system connected to an ACM desolvator (Elemental Scientific Incorporation) to enhance the signals. In this configuration, a signal of 30 cps for a solution of 1 fg/ml can be expected for actinide isotopes. The separation factors from uranium were for both americium and plutonium larger than 20,000. The interferences from uranium were corrected with tailing factors for the abundance sensitivity. The factors were measured with 10 ng/ml uranium solutions. For the mass differences 1, 2, and 3 to the mass 238 factors of 5 10^−6^, 6 10^−7^, and 1 10^−7^ were obtained, respectively. The interferences from tailing were mostly neglectable. ^241^Pu and ^241^Am concentrations were very low, and particular attention had to be directed to the washing-out of the signals. For global fallout, the ^242^Pu/^239^Pu atomic ratio was measured as 0.00385 ± 0.00076 (Ketterer et al. (2004b)). The concentration of ^242^Pu from the sample is expected to be much smaller than the added amount of ^242^Pu tracer. Thus, the concentrations of the Pu isotopes could be calculated based on the sensitivity for the ^242^Pu tracer.

For the analysis of the uranium isotope composition, the uranium eluates from the UTEVA column were diluted to 100 ng/ml ^238^U and introduced by a quartz spray chamber to the MC-ICP-MS. ^235^U and ^238^U were measured by Faraday detectors and the minor uranium isotopes by SEM detectors. The uranium tailing for the minor isotopes was corrected with a baseline subtraction. For the measurement of ^236^U, a retarding potential quadrupole lense (RPQ) was used to lower the abundance sensitivity. In order to achieve lowest detection limits for ^236^U, hydride formation with ^235^U could be reduced by introducing the solutions with an Arridus II desolvator (CETAC). Because of the higher sensitivity in this configuration, the uranium eluates had to be diluted to 10 ng/ml ^238^U. Abundance sensitivity factors ^236^I/^238^I of 1 10^–8^ were achieved. The total acquisition time per sample was 18 min. Standard sample bracketing using the IRMM-187 uranium isotope standard was applied for all measurements. IRMM-184 uranium isotope standard solutions with approximately the same uranium concentrations as in the uranium eluates were used for quality assurance. To check the accuracy of ^236^U/^238^U measurements down to ratios of 1 10^–8^, a mixture of the IRMM-187 (^236^U/^238^U = 1.2 10^−7^) and natural uranium (no ^236^U) were measured. ^236^U concentrations were calculated from the mass ratio of ^236^U/^238^U and the concentration of ^238^U.

#### Age determination of Pu

The determination of the age of a Pu material is generally based on mother-daughter relationships. The mother daughter pair ^241^Pu–^241^Am is often used for Pu age determination. The age of Pu is defined as the time since the last separation of the daughter. The age of the Pu material can be calculated from the decay equations governing the time-dependent activity concentrations of the mother and daughter radionuclides in the sample as (Nygren et al. (2007)):

1 $$Age= \left(\frac{1}{{\lambda }_{1}-{\lambda }_{2}}\right)\bullet \mathrm{ln}\left(1+\frac{\left({N}_{2}\bullet \left({\lambda }_{1}-{\lambda }_{2}\right)\right)}{{N}_{1}\bullet {\lambda }_{1}}\right)$$

In this equation, *λ*_1_ and *λ*_2_ are the decay constants of ^241^Pu and ^241^Am, respectively; and *N*_1_ and *N*_*2*_ are the amounts of ^241^Pu and ^241^Am at the time of analysis.

### Results and discussion

**Fig. 2:**
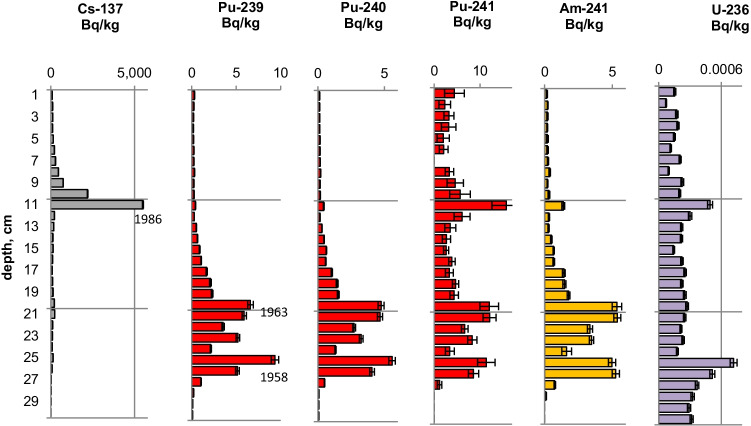
Activity concentrations (dry weight) for a sediment from Lake Lugano 2017 (LS17-SB4-2). Cs-137 and Pu-241 activities are referenced to the sampling date (04.05.2017)

**Fig. 3:**
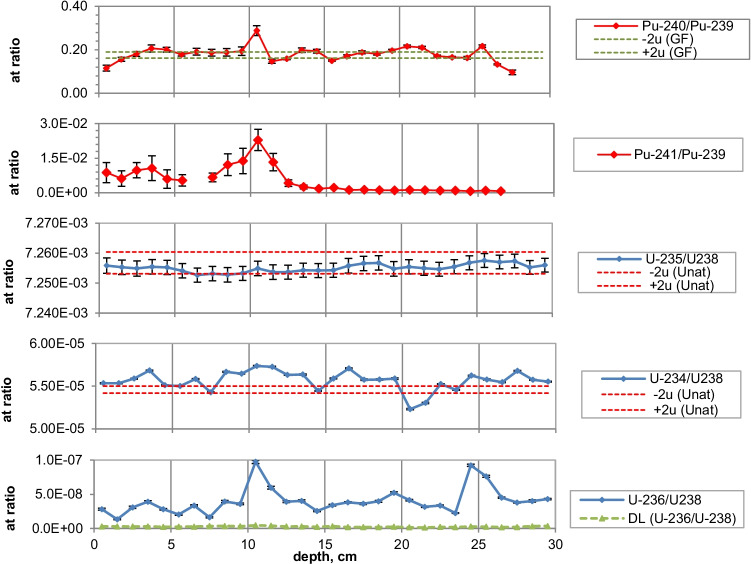
Isotope ratios in a sediment from Lake Lugano 2017 (LS17-SB4-2). Pu-241 is referenced to the sampling date

**Fig. 4:**
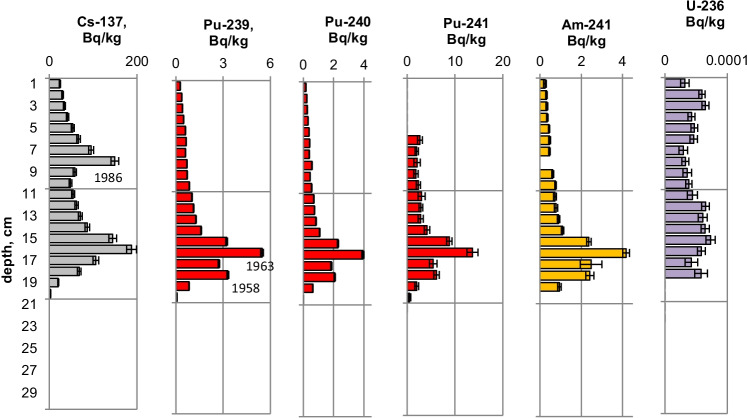
Activity concentrations (dry weight) for a sediment from Lake Brienz 2009 (BS09-LS2). Cs-137 and Pu-241 activities are referenced to the sampling date (25.05.2009)

**Fig. 5:**
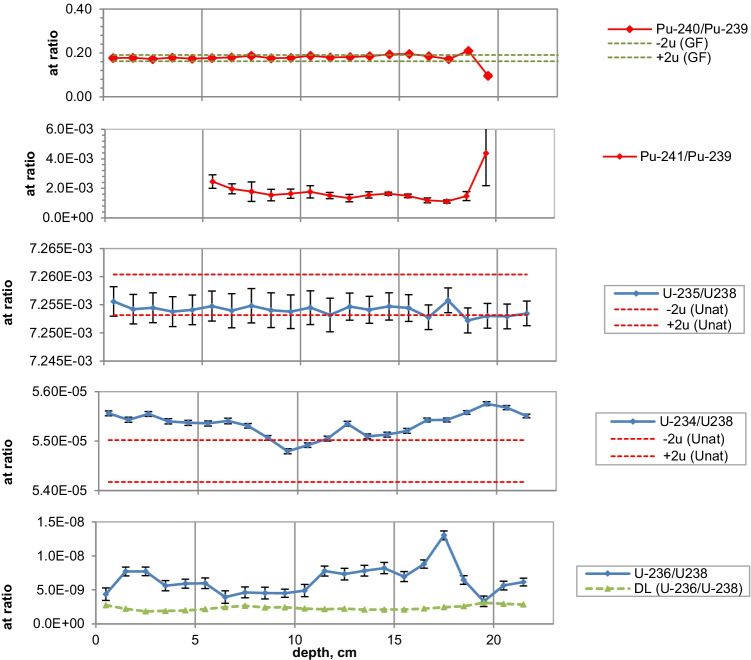
Isotope ratios in a sediment from Lake Brienz 2009 (BS09-LS2). Pu-241 is referenced to the sampling date

**Fig. 6:**
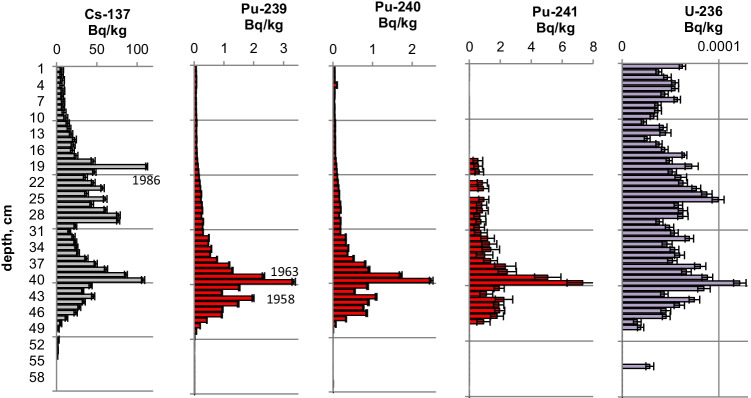
Activity concentrations (dry weight) for a sediment from Lake Biel 2013 (BL13-3d). Cs-137 and Pu-241 activities are referenced to the sampling date (18.07.2013)

**Fig. 7:**
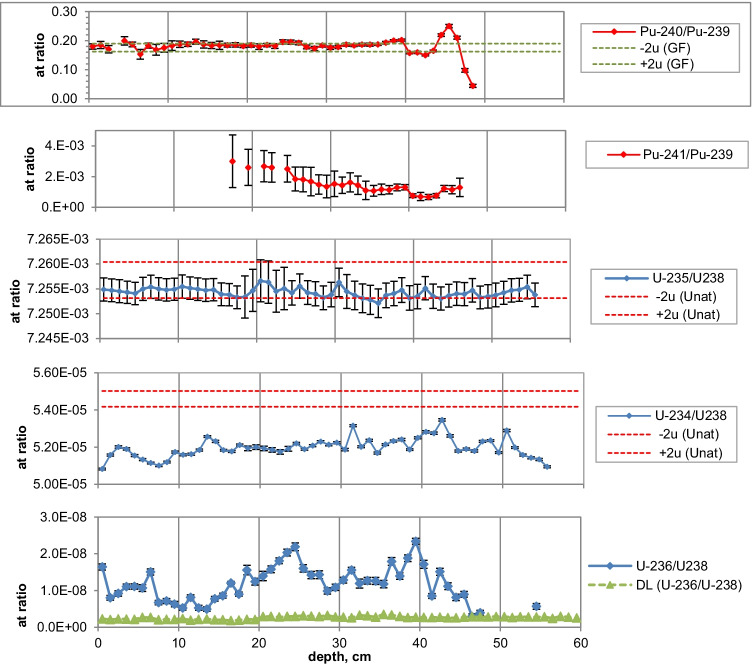
Isotope ratios in a sediment from Lake Biel 2013 (BL13-3d). Pu-241 is referenced to the sampling date

**Fig. 8:**
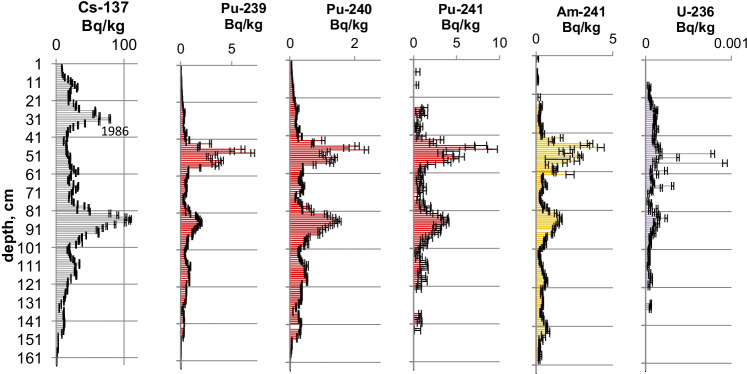
Activity concentrations (dry weight) for a sediment from Klingnau Reservoir 2017 (KL17-5). Cs-137 and Pu-241 activities are referenced to the sampling date (02.11.2017)

**Fig. 9:**
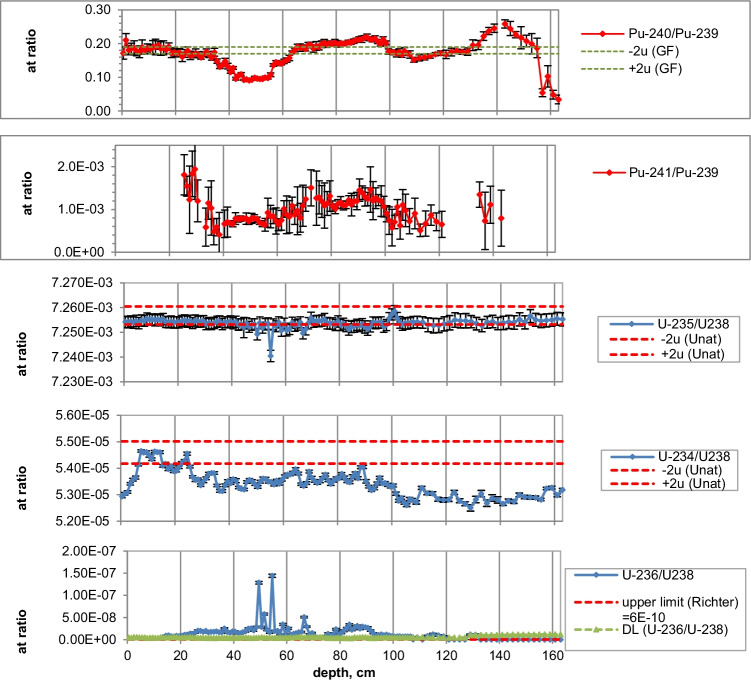
Isotope ratios in a sediment from Klingnau Reservoir 2017 (KL17-5). Pu-241 is referenced to the sampling date

Figures 2, 3, 4, 5, 6, 7, 8 and 9 show the activity concentrations of ^137^Cs, ^239^Pu, ^241^Pu, ^241^Am, ^236^U, and the plutonium and uranium isotope ratios in a sediment core from Lake Lugano, Lake Brienz, Lake Biel, and Klingnau Reservoir. The ^240^Pu/^239^Pu isotope ratio for global fallout (Ketterer et al. (2004b)) and the ^235^U/^238^U and ^234^U/^238^U isotope ratio for natural U (Richter et al. (1999)) are represented as dashed lines. The reference date for ^137^Cs, ^241^Pu and ^241^Am corresponds to the sampling date of each core.

The maximum ^137^Cs activity from the Chernobyl accident and the Pu and ^137^Cs activities associated to the 1963 global fallout maximum were well identified in sediments from all the three lakes. For the Klingnau reservoir, it is difficult to associate a peak to the NWT testing because of additional inputs of ^137^Cs and Pu isotopes from the nuclear power plants. Table 2 shows the Chernobyl and NWT inventories for ^137^Cs and ^239^Pu for the three lakes. The upper parts of the sediment cores are dominated by the Chernobyl contamination. The ^137^Cs Chernobyl inventory was calculated by summing up the area-specific activities associated with the respective peak and the area above. Only for the Lake Lugano a slightly higher plutonium concentration in the Chernobyl layer was found. The plutonium contribution of this layer was < 1 Bq/kg. The NWT inventory was calculated by summing up the area-specific activities associated with the NWT peak and the area above until the Chernobyl peak. For Lake Biel, additional ^137^Cs inputs from discharges of the nuclear power plant Mühleberg have to be considered. The contribution to the NWT inventory of the area disturbed by additional ^137^Cs inputs was calculated from the Pu concentrations by assuming a constant ^137^Cs/^239^Pu ratio for NWT input. The Chernobyl ^137^Cs was referenced to the year 1986 and the NWT ^137^Cs to the year 1963. The measured inventories correspond well to inventories calculated from surface soil samples (0–20 cm) (Meusburger et al. 2020). Because of much higher precipitation in the southern part of Switzerland in May 1986, up to 7 times more ^137^Cs was deposited in Lake Lugano compared to Lake Biel. Highest plutonium inventories were measured in sediments from Lake Brienz, corresponding to higher rainfalls in the Alpes in the time period 1952–1985.

**Table 2:** Chernobyl and NWT ^137^Cs inventories decay-corrected the year 1986 and 1963, respectively. Plutonium concentrations are dominated by global fallout, therefore only ^239^Pu NWT inventories could be calculated

	Chernobyl		NWT	
	^137^Cs kBq/m^2^	^239^Pu + ^240^Pu Bq/m^2^	^137^Cs kBq/m^2^	^239^Pu + ^240^Pu Bq/m^2^
Lake Lugano	21	< 1	8.6	128
Lake Brienz	7.5	-	17.5	255
Lake Biel	2.9	-	10.8	167

The NWT contamination of ^137^Cs and Pu isotopes in the sediment shows a double-peak structure for the cores of all three lakes. These two peaks are characteristic for the periods 1951–1958 and 1961–1962, respectively, matching the chronological order of NWT activities worldwide. The first period spans more than approximately 7 years and ends with a maximum in 1958, while the second period is shorter but more intense, with a maximum in 1962. The local minimum in between corresponds to the nuclear test moratorium for the years 1959/60 (UNSCEAR (2000)). Assuming an average delay of 1 year for the deposition of the global fallout, peak maxima are expected for the years 1958 and 1963 and a minimum for the year 1960 (Warneke et al. (2002)).

The ^240^Pu/^239^Pu atom ratios in the sediment cores varied with depth showing a similar pattern as was found in the annual grass samples from Rothamsted, UK (Warneke et al. (2002)). The records show four major distinct features that can be associated to certain time periods: (i) an increase between 1952 and 1959; (ii) a decrease between 1959 and 1961; (iii) an increase between 1961 and 1963; and (iv) a rather constant ratio from 1964 up to the present days in the cores only affected by global fallout. As the layer thickness was 1 cm, the structure was more distinct for lakes with a higher average sedimentation rate.

**Fig. 10:**
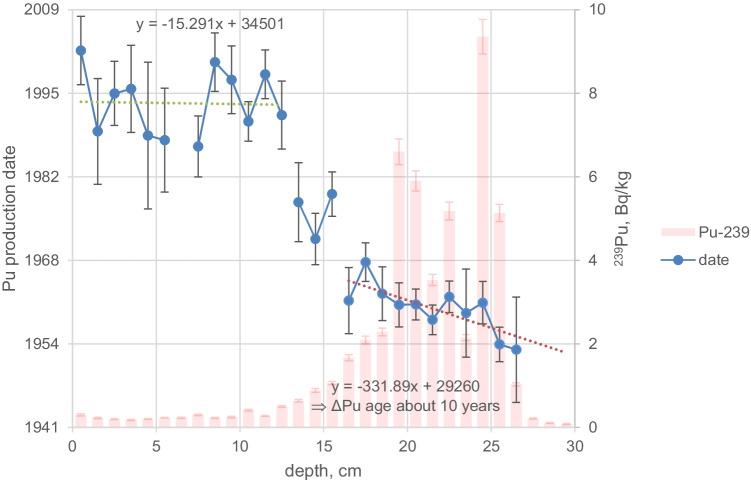
Plutonium production date and ^239^Pu activity concentrations for the sediment core Lake Lugano 2017 (LS17-SB4-2)

For Lake Lugano, a small additional plutonium input can be recognized in the sediment layers corresponding to the Chernobyl accident in 1986. For this layer, ^240^Pu/^239^Pu and ^241^Pu/^239^Pu atomic ratios (corrected for the reference year 1986) of 0.29 ± 0.02 and 0.10 ± 0.02, respectively, were measured. Jakopič et al. (2010) reported ^240^Pu/^239^Pu and ^241^Pu/^239^Pu ratios of 0.414 ± 0.004 and 0.117 ± 0.007, respectively, for plutonium particles from the Chernobyl accident. The difference of the Pu isotope ratios measured in Lake Lugano compared to those observed in Chernobyl plutonium could be explained by the additional contribution of NWT plutonium. Because of the small half-life of ^241^Pu (*t*_1/2_ = 14.3 years), about 95% of the NWT ^241^Pu has decayed meanwhile to ^241^Am. Despite the relative high uncertainties for the determination of ^241^Pu due to the very low concentrations, the ^241^Pu/^239^Pu ratio seems to be higher in the Chernobyl accident layer compared to the NWT zone (Fig. 3). Assuming that all ^241^Am originated from the decay of ^241^Pu, the Pu production date can be calculated. Figure 10 shows the calculated production dates for Pu found in Lake Lugano. The calculated production dates seem to be dominated by NWT plutonium (production dates 1953–1968) in layers deeper than 15 cm and in the upper zone by the additional plutonium input with a production date of 1994 ± 5 years. The age of plutonium is slightly increasing with depth in the NWT zone, leading to a maximal plutonium age difference of about 10 years whereas for the uppermost 13 cm, the age seems to be rather constant. The age of the plutonium should not be confused with the age of the sediment layer. The difference in the plutonium age means that the plutonium for the NWT tests originated not from the same plutonium batch, but was consistently separated during the test period. In the upper most 13 cm, the plutonium originated from the run-off of the global-fallout plutonium and not from direct inputs. The age of this run-off plutonium is expected to be constant. The calculated age of the plutonium seems to be younger than expected from the run-off of the bulk of GF plutonium. The reason could be the influence of additional non-global fallout plutonium from Chernobyl. In a nuclear reactor ^241^Pu is produced. The influence of the produced ^241^Am in the nuclear fuel during the run-time of the reactor is small. Hence, the calculated age refers rather to the release of the plutonium from the reactor than to the production date of the plutonium.

**Fig. 11:**
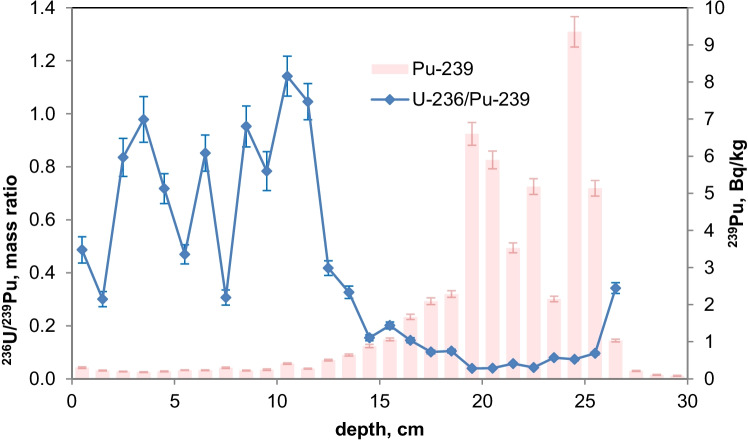
Mass ratio of ^236^U/^23^9Pu and ^239^Pu activitiy concentrations for the sediment of Lake Lugano 2017 (LS17-SB4-2)

**Fig. 12:**
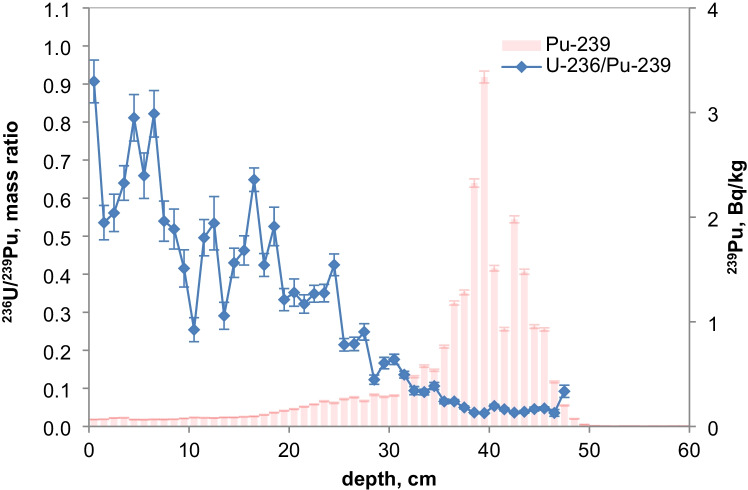
Mass ratio of ^236^U/^239^Pu and ^239^Pu activity concentrations for the sediment of Lake Biel 2013 (BL13-3d)

As the ^236^U concentrations were very close to the detection limits, the uncertainties of the ratios of ^236^U/^238^U and the calculated ^236^U concentration might be higher because of the difficulties with baseline (uranium tailing) corrections. However, a small increase of the ^236^U/^238^U ratio can be recognized for the NWT zone in all three lakes and, for Lake Lugano, a further distinct increase in the Chernobyl layer. For Lake Biel, an increase of the ^236^U/^238^U ratio occurs in the zone where additional ^137^Cs from discharges of the nuclear power plant Mühleberg was found (Fig. 6). Figures 11 and 12 show the mass ratio of ^236^U/^239^Pu for the sediments for Lake Lugano and Lake Biel. For both lakes, ratios < 0.1 were measured for the NWT zone. For the time period before 1980, the ^236^U/^238^U ratios show a trend towards higher ratios up to 0.8–1 with rather high variations. The ^236^U/^238^U ratios seem to increase continuously towards the top of the sediment for Lake Biel whereas for Lake Lugano the increase is more abrupt. The reason for the change of the ratio ^236^U/^239^Pu could be non-global fallout sources or a higher mobility of uranium compared to plutonium. Srncik et al. (2011) reported ^236^U/^239^Pu ratio in soil profiles in a similar range from 0.04 to 0.78, and > 3 in the topmost layer (0–2 cm). Sakaguchi et al. (2009) reported ^236^U/^238^U atomic ratios for the global fallout in a close range between 0.212 and 0.252.

Natural ^235^U/^238^U ratios were measured in the sediments of the three lakes. For Lake Lugano and Lake Brienz, mostly enriched ^234^U/^238^U ratios and for Lake Biel, depleted ^234^U/^238^U ratios were measured. Depleted ^234^U/^238^U ratios (activity ratio < 1) in the sediments can be explained by the alpha recoil effect, which leads to activity ratios > 1 in the water. The reason for activity ratios > 1 can be sorption and precipitation effects of uranium.

The interpretation of the contamination of the Klingnau Reservoir is far more difficult, as there are additional inputs from the nuclear power stations Mühleberg, Beznau, and the nuclear facility Paul Scherer Institute. An additional plutonium input is of special interest. The ^240^Pu/^239^Pu isotope ratio is distinctly lower than expected for the global fallout plutonium. The calculated Pu production date (1952–1958) in these layers is older than the age of the sediment from the age-depth relation (1973–1979). The reason could be that Pu was stored and discharged some years later. The measurement and modeling of a few different radionuclide concentrations, and their activity ratios helped to find out the origin of the radioactive contamination (Klemt et al. (2021)).

## Conclusions

The measurement of radioactive nuclides in sediment cores can provide a good overview of the regional radioactive contamination. The Aare river system, with its two nuclear power plants on the banks of the river and its intermediate lakes and reservoir, provide the unique chance to analyze the contribution of radioactive contamination. In order to detect non-global fallout sources, an accurate knowledge of the background contamination from the global fallout and the Chernobyl accident is important. Depth profiles of nuclide concentrations and isotope ratios are extremely valuable for investigations of the origin of the radioactive material. Our study supports the use of the time features observed in the ^240^Pu/^239^Pu atom ratio, conjointly with the Pu activity maximum, as time markers for validating sediment chronologies. Age determinations defined as the production date of the radioactive contamination and depth-age relation of the sediment layers can also provide valuable information about the origin of the radioactive contamination. Isotope ratios in environmental samples can also be influenced by different mobility of the nuclides and has to be considered for the interpretation of the influence of different radioactive sources.

## Data Availability

All the data are stored in the figures of the word-file (CH lakes Figures_v2).
